# Radioprotective Efficacy of Jujube Aqueous Extract and Arbutin: Synergistic Antioxidant and Anti‐Apoptotic Mechanisms in Radiation Injury

**DOI:** 10.1002/fsn3.71073

**Published:** 2025-10-23

**Authors:** Liping Wang, Hui Yang, Xuchen Zhou, Yufen Zhao, Long Li, Ning Wang

**Affiliations:** ^1^ Institute of Drug Discovery Technology Ningbo University Ningbo China; ^2^ Qian Xuesen Collaborative Research Center of Astrochemistry and Space Life Sciences, Institute of Drug Discovery Technology Ningbo University Ningbo China; ^3^ Department of Chemical Biology, College of Chemistry and Chemical Engineering Xiamen University Xiamen China; ^4^ Key Laboratory of Bioorganic Phosphorus Chemistry and Chemical Biology (Ministry of Education), Department of Chemistry Tsinghua University Beijing China

**Keywords:** antioxidant, arbutin, jujube aqueous extract, lung tissue, radiation

## Abstract

*Ziziphus jujuba* Mill., documented in *Shennong Bencao Jing*, demonstrates medicinal agents and enhanced longevity through sustained consumption. Yet their specific medicinal components and mechanisms of action require further exploration. In light of increasing radiation exposure from medical procedures, environmental hazards, and aerospace activities, this study aims to elucidate the radioprotective mechanisms of jujube aqueous extract (JAE). JAE effects were tested in UVC‐irradiated A549 and HaCaT cells and x‐ray‐exposed mice. Cell viability, oxidative stress markers, hematological and histopathological parameters, and proteomic profiles were analyzed. Key apoptotic proteins and the core bioactive compound were identified. JAE demonstrated antioxidant activity in vitro, effectively scavenging free radicals (*p* < 0.01). In C57BL/6 mice subjected to 6 Gy x‐ray irradiation, JAE significantly alleviated lymphocytopenia (*p* < 0.05, 74.31% alleviation), attenuated lung damage, and mitigated oxidative stress by enhancing SOD (41.70% increase) and CAT (10.43% increase) activities while suppressing MDA levels (13.59% decrease, all *p* < 0.05). Proteomic profiling further identified 58 apoptosis‐associated proteins, demonstrating modulation of apoptotic pathways through upregulation of anti‐apoptotic Bcl‐XL and downregulation of pro‐apoptotic Bax, Bik, and cytochrome c (*p* < 0.05). In A549 and HaCaT cells, JAE enhanced viability (*p* < 0.05, 37.06% and 14.08% increase), reduced ROS, and restored redox homeostasis (*p* < 0.05). Arbutin was identified as a key bioactive compound in JAE, conferring radioprotection by anti‐apoptotic, anti‐inflammatory, and stabilizing mitochondrial function. By combining proteomics with functional validation, this study reveals JAE's radioprotective mechanism via apoptosis modulation and antioxidant enhancement, highlighting arbutin as a key mediator. These findings provide a pharmacological foundation for developing jujube‐based adjuvants in radiation oncology and aerospace medicine.

## Graphical abstract

JAE demonstrates potent antioxidant activity and protects against radiation‐induced lung injury. Proteomic reveals JAE modulates apoptosis and oxidative stress pathways for radioprotection. JAE demonstrates broad cellular protection against UVC damage in lung and skin cell models. Arbutin is identified as the key bioactive compound in JAE mitigating radiation injury. Arbutin dually inhibits apoptosis and pyroptosis.

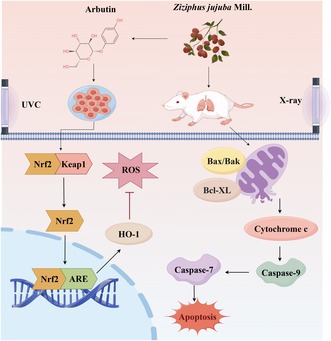

**Abbreviations**
6G: 6‐Gingerol
ABTS^+^
: 2,2'‐Azino‐bis(3‐ethylbenzothiazoline‐6‐sulfonic acid) radical cation
AT: Arbutin
CAT: Catalase
CBC: Complete blood count
CCK‐8: Cell Counting Kit‐8
CS: Cytisine
CT: Computed tomography
DA: Dihydrocaffeic acid
DPPH: 2,2‐Diphenyl‐1‐picrylhydrazyl radical
ELISA: Enzyme‐linked immunosorbent assay
GA: Gallic acid
GSH/GSSG: Glutathione (reduced)/Glutathione disulfide (oxidized)
H&E: Hematoxylin and eosin
IL‐1β: Interleukin‐1β
IQ: Isoquercitrin
JAE: Jujube aqueous extract
KA: Kojic acid
LC–MS: Liquid Chromatography‐Mass Spectrometry
MDA: Malondialdehyde
Nrf2: Nuclear factor erythroid 2‐related factor 2
O_2_^−^
: Superoxide anion radical
‐OH: Hydroxyl radical
PBS: Phosphate‐buffered saline
PFA: Paraformaldehyde
PG: Propyl gallate
RH: Rutin
ROS: Reactive oxygen species
SOD: Superoxide dismutase
SPF: Specific pathogen‐free
SQ: Esculetin (Scopoletin derivative)
TCM: Traditional Chinese Medicine
UVC: Ultraviolet‐C radiation

## 1 Introduction

Radiation exposure pervades modern environments through three primary domains: medical diagnostics (notably computed tomography (CT) scans delivering typical doses below 10 mSv per procedure—approximately 100 times greater than the 0.1 mSv exposure from conventional chest x‐rays), ubiquitous consumer electronics operating within international safety standards, and naturally occurring solar ultraviolet (UV) radiation, particularly in regions with elevated UV indices approaching extreme levels near equatorial zones or at high altitudes (Lin 2010; Murphy 2009). Collectively, these cumulative sources elevate global health risks (Czégény et al. 2016; Tang et al. 2024). Space exploration programs face significant radiation protection challenges; astronauts encounter complex radiation environments during missions. Galactic Cosmic Rays and Solar Particle Events contribute to acute high‐dose exposures during solar flares, while chronic low‐dose radiation persists during extended deep‐space travel (Cucinotta et al. 2021). Radiation toxicity manifests via dual mechanisms: direct ionization induces critical lesions like double‐strand DNA breaks at > 0.5 Gy, while indirect oxidative stress generates reactive oxygen species (ROS) (Forenzo and Larsen 2024; Li et al. 1998). These ROS drive cytotoxicity through oxidative stress, DNA fragmentation, protein denaturation, and lipid peroxidation, ultimately triggering apoptosis. Notably, radiotherapy relies on this ROS‐mediated cytotoxicity for tumor eradication, but cancer cells often exhibit dysregulated redox buffering capacity that compromises treatment efficacy. Furthermore, post‐irradiation inflammatory homeostasis fluctuates dynamically between pro‐ and anti‐inflammatory states. These complexities underscore the need for agents that simultaneously protect healthy tissues while sensitizing malignancies—a dual role increasingly associated with natural antioxidants (Uguz et al. 2024). ROS‐driven damage triggers apoptosis in radiosensitive tissues; the effective (mean dose required to reduce cell survival to 37%) for human cells averages approximately 3 Gy, ultimately provoking immune collapse, bone marrow failure, and multi‐organ degeneration (Dainiak 2002). These cascading effects underscore the imperative to address radiation‐associated morbidity across terrestrial and extraterrestrial environments. Radiation, as a pervasive environmental stressor, necessitates safe and sustainable radioprotective solutions. While synthetic agents targeting isolated molecular pathways are often limited by toxicity and transient efficacy (Kamran et al. 2016), natural products with multi‐target capacity and low adverse effects have emerged as next‐generation candidates (Hu et al. 2024). Specifically, flavonoids and polyphenols—abundant in dietary botanicals—exemplify this promise by concurrently scavenging reactive oxygen species (ROS), modulating antioxidant enzymes (e.g., superoxide dismutase, glutathione reductase), and suppressing pro‐inflammatory cascades, all with minimal toxicity (Avcı et al. 2025; Özdemir and Demir 2025). These compounds leverage synergistic mechanisms to enhance cellular resilience against oxidative stress, a hallmark of radiation injury, while avoiding the off‐target effects common to synthetic agents.

*Ziziphus jujuba* Mill. (Jujube) stands out as a medicinal‐food homologous substance for over 2000 years, has been extolled in ancient texts such as “Shennong Bencao Jing” for its ability to “tonify the middle *jiao*, replenish qi, and harmonize the blood”, as well as in “*Bencao Gangmu*” for its role in “alleviating palpitations and prolonging vitality”. These historical claims align with modern pharmacological insights: Jujube contains abundant bioactive metabolites such as polysaccharides, polyphenols (flavonoids, anthocyanins), terpenoids, and alkaloids (Ghasemzadeh Rahbardar et al. 2024). It confers multifaceted pharmacological activities, including hematopoietic (Chen and Tsim 2020), anti‐oxidant (Lu et al. 2021), anti‐inflammatory (Goyal et al. 2011), anti‐microbial (Mehreen et al. 2016), anti‐asthmatic (Ninave and Patil 2019), immunomodulatory (Nergard et al. 2005), neuroprotective (Zhu et al. 2024), and anti‐anxiety effects (Peng et al. 2000). Arbutin demonstrates radioprotection in mice against megavoltage therapeutic x‐rays and demonstrates radioprotective efficacy through DNA damage prevention (Nadi et al. 2019; Wu et al. 2014). Specific jujube‐derived caffeic acid reverses UVC‐irradiated lymphocyte damage (Prasad et al. 2009), epicatechin alleviates radiation‐associated hepatic oxidative stress (Sinha et al. 2012), and rutin/quercetin synergistically neutralize gamma radiation toxicity (Patil et al. 2013). Studies suggest that bioactive compounds in *Ziziphus jujuba* Mill., such as flavonoids and polysaccharides, contribute to enhancing the body's antioxidant capacity and protecting cells from damage (Liu et al. 2025). While numerous natural products, including flavonoids and polysaccharides, have demonstrated radioprotective potential through mechanisms such as antioxidant activity, DNA damage repair, and immune modulation (Smith et al. 2017), research specifically on *Ziziphus jujuba* Mill. remains relatively scarce and incomplete. Existing literature on jujube has primarily focused on its nutritional value, antioxidant properties, or its use involving irradiation. A critical research gap exists in the systematic evaluation of its efficacy as a *prophylactic* radioprotective agent against ionizing radiation‐induced damage in mammalian systems, particularly concerning its effects on hematopoiesis, pulmonary injury, and the precise molecular mechanisms involved.

This study employed an integrated approach to investigate the radioprotective mechanisms of jujube aqueous extract (JAE), prioritizing in vivo evaluation followed by in vitro validation. The systemic protective efficacy of JAE was first examined using an x‐ray‐exposed murine model, focusing on its ability to mitigate radiation‐induced damage at the organismal level. Subsequently, in vitro models utilizing UVC‐irradiated A549 lung epithelial and HaCaT keratinocyte cells were implemented to dissect cellular and molecular mechanisms. The research systematically explored JAE's antioxidant capacity in scavenging radiation‐generated ROS, its modulation of mitochondrial‐mediated anti‐apoptotic pathways, and the critical role of arbutin as the principal bioactive constituent. Proteomic profiling coupled with functional validation revealed JAE's multi‐target therapeutic potential, providing mechanistic insights into its dual action in cellular protection and systemic radioprotection, thereby advancing the development of nature‐derived radiation countermeasures.

## 2 Materials and Methods

### 2.1 Preparation of JAE

Fresh jujube fruits (Xinjiang Fruit Industry Co., China) were washed with deionized water (Heal Force Bio‐Tech, China) to remove surface impurities and air‐dried at 25°C. Dried fruits (500 g) were soaked in 1 L of ultrapure water for 30 min at room temperature. The hydrated mixture underwent high‐pressure steam sterilization using an autoclave (Shunzhi, Shandong, China), followed by natural cooling to 25°C. After sterilization, the softened fruits were manually peeled to remove pits and skins. The pulp was homogenized into a paste and centrifuged (Eppendorf, Hamburg, Germany) at 8000 × g for 15 min at 4°C. The supernatant was collected and concentrated to a solid residue via rotary evaporation (Thermo Fisher Scientific, USA), weighed to calculate extraction yield, aliquoted into sterile amber vials, and stored at −80°C under light‐protected conditions until further analysis.

### 2.2 Cell Culture and UVC Cell Assay

HaCaT (human keratinocyte) cells (Starfish Biotechnology, China) and A549 (lung epithelial) cells (Shanghai Cell Bank, China) were maintained in DMEM and F‐12 K medium (both with 10% FBS) at 37°C, 5% CO_2_, routinely passaged with 0.25% trypsin–EDTA every 2–3 days.

Cells were seeded and allowed to reach 50%–60% confluence. After washing with phosphate‐buffered saline (PBS, Biotech Co., China), cells were exposed to UVC irradiation (non‐irradiated controls were maintained under identical conditions). Following irradiation, fresh culture medium was replenished, and cells were returned to standard incubation conditions. Cell viability was quantitatively assessed at 24 h intervals using the Cell Counting Kit‐8 (CCK‐8) assay (APExBIO, USA), with absorbance measured at 450 nm using a microplate reader (Hu et al. 2021).

### 2.3 Animals and Model

Male BALB/c mice (*n* = 30), aged 6–8 weeks and weighing 21–25 g, were purchased from Vital River Laboratory Animal Technology Co. Ltd. (Beijing, China). The mice were acclimatized for 1 week in a specific pathogen‐free (SPF) environment under controlled conditions: constant temperature of 21°C–23°C, humidity of 50%–60%, and a 12‐h light/dark cycle, with ad libitum access to food and water. After acclimatization, the mice were divided into experimental groups and continuously administered treatments for 14 days at a dose of 2500 mg/kg. Six hours after the final dose, the animals were exposed to 6 Gy X‐irradiation.

All animal experiments were approved by the Animal Ethics Committee of Huitong (Approval No. HTDW‐202302005) and conducted in strict compliance with national guidelines for the ethical use of animals in scientific research.

### 2.4 Animals Sample Collection and Preparation

Following euthanasia, whole blood was collected via cardiac puncture into EDTA‐2Na‐coated anticoagulant tubes to preserve plasma integrity, with a portion immediately processed for complete blood count (CBC) analysis. The remaining anticoagulated blood was centrifuged to isolate plasma, which was aliquoted and stored at −80°C for downstream assays. Concurrently, organs (heart, liver, spleen, kidneys, and lungs) were meticulously excised, rinsed in PBS, blotted dry, and weighed for organ index calculation using the formula:

$$\text{Organ Index}\;\left(\%\right)=\left[\text{Organ Weight}\;\left(g\right)/\text{Final Body Weight}\;\left(g\right)\right]\times 100.$$

Lung tissues were processed in parallel: one segment was fixed in 4% paraformaldehyde (PFA) at 4°C for 24 h, embedded in paraffin, and sectioned into 5–10 μm slices using a microtome for histopathology, while the remaining tissue was snap‐frozen in liquid nitrogen. All flash‐frozen tissues and plasma aliquots were maintained at −80°C until biochemical or molecular analyses. Body weights were recorded pre‐irradiation (6 h prior to x‐ray exposure) and post‐treatment to normalize organ indices.

### 2.5 Hematoxylin and Eosin (H&E) Staining

Paraformaldehyde‐fixed lung tissue sections were dehydrated through a graded ethanol series (70%, 80%, 90%, and 100%), cleared in xylene, and embedded in paraffin. Sections (5–10 μm thick) were mounted on glass slides, deparaffinized in xylene, and rehydrated through a descending ethanol gradient. H&E staining was performed: sections were stained with hematoxylin for 5 min, rinsed in tap water, differentiated in 1% acid alcohol, and counterstained with eosin for 2 min (He et al. 2025). After dehydration in ethanol and clearing in xylene, slides were coverslipped with neutral resin and examined under a light microscope for morphological assessment.

### 2.6 In Vitro Anti‐Oxidant Assays

JAE, arbutin, and vitamin C (VC, positive control) were prepared and incubated with reaction systems containing DPPH, 2,2′‐Azino‐bis (3‐ethylbenzothiazoline‐6‐sulfonic acid) radical cation (ABTS^+^), hydroxyl radical (‐OH), or superoxide anion radical (O_2_^−^) following standardized protocols. Absorbance values were recorded using a microplate reader at characteristic wavelengths (517 nm for 2,2‐Diphenyl‐1‐picrylhydrazyl radical (DPPH), 734 nm for ABTS^+^, 510 nm for ‐OH, and 325 nm for O_2_^−^) (Rumpf et al. 2023). Data were normalized against negative and blank controls to quantify radical scavenging activity.

### 2.7 The Assessment of Oxidative Stress

Samples were homogenized in ice‐cold strong RIPA buffer (1:10 w/v), centrifuged at 12,000 × g for 15 min (4°C), and supernatants were collected. Protein concentrations were determined using the Bicinchoninic Acid Assay (BCA, solarbio, China) method (Pierce Kit). Malondialdehyde (MDA) content, superoxide dismutase (SOD), catalase (CAT) activities, ROS levels, and Glutathione/Glutathione disulfide (GSH/GSSG) ratios were measured using commercial assay kits (Nanjing Jiancheng Bioengineering Institute) (Cui et al. 2022). All steps were performed under reduced light, with triplicate measurements to ensure reproducibility.

### 2.8 UHPLC–MS Analysis

Separation was carried out on a Waters X Bridge C18 column (3.0 × 150 mm, 1.7 μm particle size) maintained at 30°C. A binary mobile phase system was employed, consisting of (A) 5% acetonitrile in water containing 0.1% formic acid (v/v) and (B) 100% acetonitrile. The gradient elution profile was programmed as follows: 0–3 min, 0% B (isocratic); 3–15 min, linear ramp to 40% B; 15–18 min, stepwise increase to 90% B; 18–24 min, return to initial conditions (0% B). A 6 min column re‐equilibration phase (24–30 min) ensured baseline stability, yielding a total runtime of 30 min. The flow rate was fixed at 0.30 mL/min, and the injection volume was 5 μL. Analytes were monitored at 259 nm using a photodiode array (PDA) detector.

Mass detection was performed in positive ionization mode (ESI+) via a heated electrospray ionization (HESI) source coupled to a high‐resolution mass spectrometer. Source parameters were optimized as follows: spray voltage, 3.5 kV; sheath gas flow rate, 40 arb; auxiliary gas flow rate, 10 arb; capillary temperature, 380°C; auxiliary gas heater temperature, 400°C. Full‐scan spectra were acquired in the range of m/z 50–750 with a dwell time of 100 ms per fragmentation pathway.

### 2.9 Proteomics Analysis

Protein samples extracted from lung tissue were processed using the MicroFASP method with Ultracel‐10 membrane‐based 10 kDa centrifugal filters. Transferring 100 μg of protein (quantified via BCA assay) into labeled EP tubes, the samples were reduced with 5 mM TCEP in 50 mM ABC buffer (37°C, 1 h, dark) and alkylation with 10 mM iodoacetamide (room temperature, 25 min, dark). Pre‐washed centrifugal filters (50 mM ABC buffer, 14,000 × g, 10 min) were loaded with reduced/alkylated samples for three centrifugation cycles (14,000 × g, 15 min each). Trypsin digestion was conducted at 37°C with a stepwise enzyme‐to‐protein ratio (1:100 for 4 h; 1:50 overnight). Resulting peptides were eluted, vacuum‐dried, and stored at −80°C, then reconstituted in 0.1% formic acid prior to Liquid Chromatography‐Mass Spectrometry (LC–MS/MS) analysis (Zhang et al. 2021). For proteolysis optimization, proteins were acetone‐precipitated (1:5 sample‐to‐acetone ratio, −20°C, overnight) post‐reduction/alkylation. Pellets were washed with 90% acetone (−20°C), air‐dried, and digested with trypsin (Promega, 1:50 ratio in 100 mM TEAB, 37°C, overnight). Desalting via C18 spin columns preceded lyophilization after peptide quantification.

Nano ultra‐high‐performance liquid chromatography–tandem mass spectrometry (nano UHPLC–MS/MS) analysis was performed using an Orbitrap Fusion Lumos Tribrid Mass Spectrometer (Thermo Fisher Scientific). Peptides were separated on an Acclaim PepMap C18 column (Thermo Fisher Scientific) with a 60‐min gradient from 2% to 35% mobile phase B (0.1% formic acid in acetonitrile) at a flow rate of 250 nL/min. Data‐independent acquisition (DIA) parameters included MS1 scans (350–1200 m/z, 120,000 resolution) and HCD‐MS/MS (50,000 resolution, 33% collision energy) across 60 variable windows. Raw data were analyzed using Proteome Discoverer 2.3 (Thermo Fisher Scientific) against the UniProt *Mus musculus* database with the following parameters: precursor mass tolerance of 10 ppm, fragment mass tolerance of 0.02 Da, dynamic modifications for oxidation (+15.995 Da) of methionine and N‐terminal acetylation (+42.011 Da), and static carbamidomethylation (+57.021 Da) of cysteine. Peptide‐spectrum matches (PSMs) were filtered at a false discovery rate (FDR) of < 1% using Percolator. For DIA quantification, Spectronaut (Biognosys AG, version 17) was employed with default settings, and differentially expressed proteins were identified using a Student's *t*‐test with a *q*‐value threshold of < 0.05 and |log2(fold change)| ≥ 2. Quality control included monitoring peptide intensity distributions (CV < 20% across replicates) (Vuolo et al. 2018).

### 2.10 Network Pharmacology Analysis

Proteome Discoverer 2.1 was employed to analyze protein peptide mass spectrometry data, with the “Protein Database” configured to *Mus musculus* (house mouse). Additional parameters were optimized according to the user manual to ensure the accuracy and coverage of peptide identification, ultimately generating peptide‐gene mapping for the sample proteome. Differential proteins between the control and treatment groups were screened using stringent thresholds (*q*‐value ≤ 0.05 and |log2FC| ≥ 2).

A Venn diagram tool was utilized to visualize overlapping targets between jujube and radiation‐induced disease. The protein–protein interaction (PPI) network of differentially expressed proteins was constructed using the STRING database (v11.0; https://www.string‐db.org/) with a minimum required interaction score set to “high confidence” (0.700). The resulting network was imported into Cytoscape (v3.7.2) for visualization and analysis. The MCODE plugin was used to identify highly interconnected clusters with the following parameters: Degree Cutoff = 2, Node Score Cutoff = 0.2, K‐Core = 2, and Max. Depth = 100. Functional enrichment analysis was carried out with DAVID, and bar plots were generated using Perl scripts for visualization. Enrichment analysis of Gene Ontology (GO; http://www.geneontology.org) and KEGG pathways (http://www.genome.jp/kegg/) for the “drug‐disease” intersection targets was conducted and visualized via the Microbioinformatics platform (Isıyel et al. 2025). This integrative approach aimed to dissect jujube anti‐radiation effects by identifying key biological processes (BP), cellular components (CC), molecular functions (MF), and signaling pathways, thereby elucidating its potential biological functions and mechanistic pathways.

### 2.11 Western Blot

Protein samples were quantified using a BCA assay, normalized, and denatured by heating in a metal bath at 100°C for 5 min. Equal protein amounts (10–30 μg) were resolved on a 7%–12.5% SDS‐PAGE gel and transferred to a PVDF membrane via wet transfer (100 V, 90 min). The membrane was blocked with 5% non‐fat milk in TBST for 1 h at room temperature (RT), followed by overnight incubation at 4°C with primary antibodies diluted in blocking buffer: Bcl‐XL (1:5000), Bik (1:5000), Bax (1:5000), Cytc (1:1000), Caspase9/p35/p10 (1:1000), Caspase7/p20 (1:1000), Cleaved PARP (1:2000), β‐actin (1:10,000), Bcl‐2 (1:1000) (all from Proteintech); phospho‐γH2AX (1:1000). After three TBST washes (10 min each), the membrane was incubated with HRP‐conjugated secondary antibody (1:5000 in TBST) for 1 h at RT, washed again, and developed using an ECL substrate. Chemiluminescent signals were captured with a digital imaging system, and band intensities were quantified using ImageJ software, with β‐actin serving as the loading control for normalization and relative expression analysis.

### 2.12 Cell Cycle and Apoptosis Analysis

After harvesting, cells were washed with PBS (Biotech Co., China) and fixed in 70% ice‐cold ethanol overnight at 4°C. The cell pellet was collected by centrifugation (1000 × g, 5 min) and resuspended in a staining solution containing propidium iodide (PI, 50 μg/mL) and RNase A (100 μg/mL). The suspension was incubated at 37°C for 30 min in the dark, followed by storage at 4°C protected from light until analysis. Flow cytometry was performed using a CytoFlex S Flow Cytometer (Beckman Coulter, USA), with 10,000 events recorded per sample. DNA content distribution across cell cycle phases (G0/G1, S, G2/M) and the sub‐G0 apoptotic population was analyzed using CytoExpert software.

For apoptosis assessment, treated cells were gently resuspended in 1× Annexin V binding buffer (Beyotime, China) and dual‐stained with Annexin V‐FITC (5 μL) and propidium iodide (PI, 10 μL) for 10–20 min at room temperature in the dark (Yang et al. 2025). Samples were immediately transferred to ice and analyzed using a CytoFlex S Flow Cytometer (Beckman Coulter, USA). This method distinguishes early apoptotic cells (Annexin V‐FITC+/PI−) from late apoptotic/necrotic cells (Annexin V‐FITC+/PI+), ensuring precise quantification of apoptotic populations.

### 2.13 JC‐1 Mitochondrial Depolarization Assay

After harvesting, stained with JC‐1 working solution at 37°C for 20 min, and washed twice with 1× JC‐1 staining buffer (Beyotime, China). Fluorescence was analyzed using a SpectraMax Paradigm Multi‐Mode Microplate Reader (Molecular Devices, USA). The 590/530 nm ratio was calculated to assess mitochondrial depolarization. A decreased ratio indicates loss of mitochondrial membrane potential (ΔΨm), a marker of early apoptosis.

### 2.14 Inflammatory Factor Detection

Measure Caspase 1 activity using the Beyotime Caspase 1 Activity Assay Kit, first generate a pNA standard curve by incubating serially diluted pNA standards (0–20 μM) with reaction buffer containing Ac‐YVAD‐pNA substrate (1:1 ratio) at 37°C for 2 h in the dark, followed by A_405_ measurement on a microplate reader. For cell samples, lyse treated cells in ice‐cold lysis buffer, centrifuge (16,000 × g, 15 min, 4°C), and mix 50 μL supernatant with 50 μL substrate‐containing buffer; after 2 h incubation (37°C, dark), measure A_405_ and calculate Caspase 1 activity (nmoL pNA/min/mg protein) using the standard curve.

For Interleukin‐1β (IL‐1β) quantification using an ELISA kit, prepare a standard curve (0–500 pg/mL) by adding IL‐1β standards to the pre‐coated plate. Collect clarified cell supernatant or lysate by centrifugation (1000 × g, 10 min, 4°C), and add 100 μL of each sample or standard to the plate in triplicate. Incubate at 37°C for 90 min, wash thoroughly, and sequentially add biotinylated detection antibody (60 min, 37°C), HRP‐streptavidin (30 min, 37°C), and TMB substrate (15–20 min, dark). Stop the reaction and measure absorbance at 450 nm to determine IL‐1β concentration. Include controls (blank, untreated, and stimulated) and normalize IL‐1β levels to cell count or total protein concentration (BCA assay) for accurate quantification.

### 2.15 Statistical Analysis of Data

All statistical analyses were conducted using GraphPad Prism 9.5.1, with quantitative data expressed as mean ± standard deviation (SD). One‐way ANOVA with post hoc Tukey test (for multiple comparisons) or unpaired Student *t*‐test (for pairwise comparisons) was applied to determine intergroup differences. *p*‐value < 0.05 was considered statistically significant, with exact *p*‐values annotated in figures (ns: *p* ≥ 0.05, *p < 0.05, **p < 0.01, ***p < 0.001).

## 3 Results

### 3.1 In Vitro Antioxidant Activity of JAE

**FIGURE 1:**
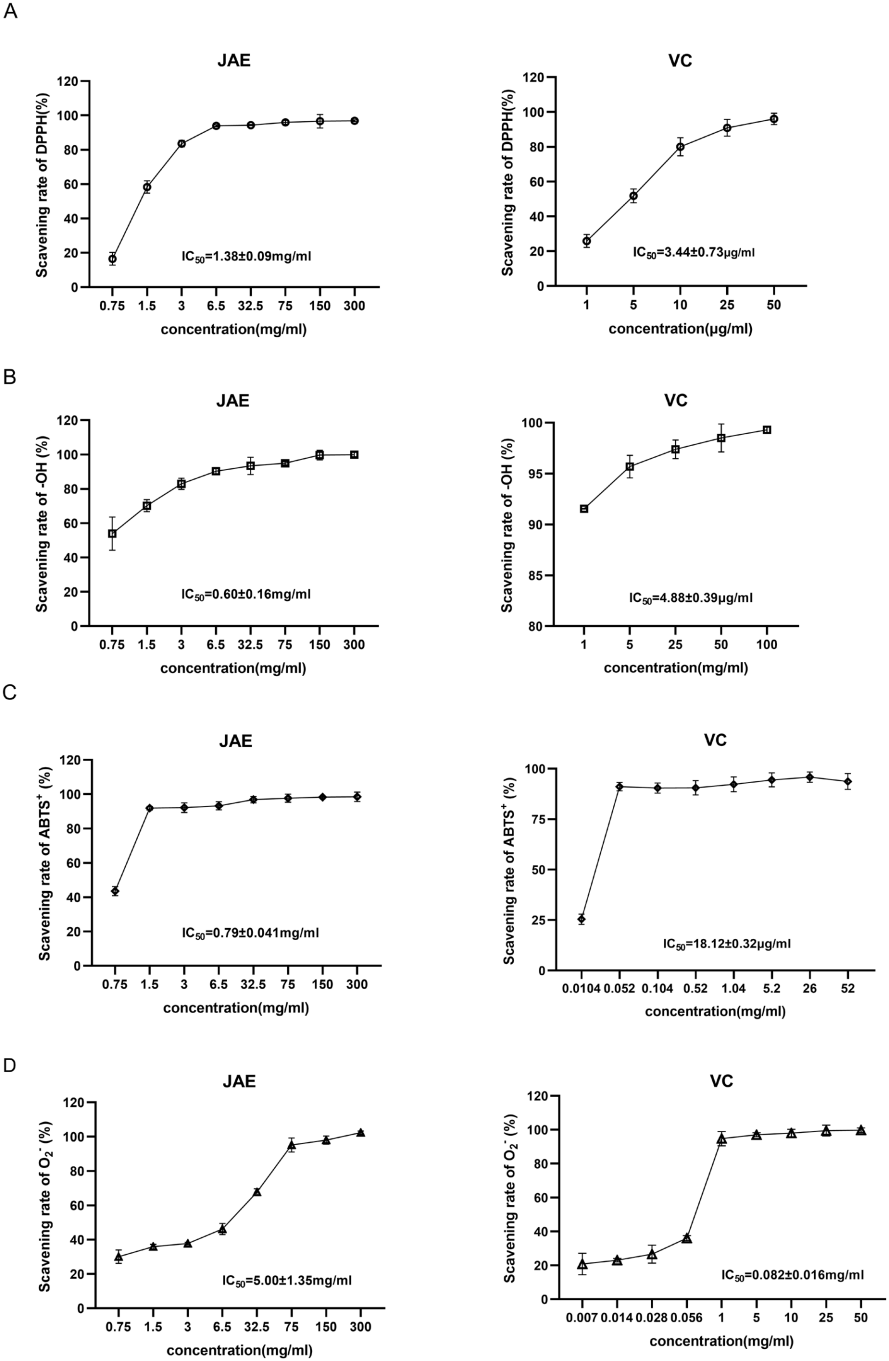
Antioxidant activities of JAE and VC against different radicals. (A–D) DPPH radical scavenging test; (B) ‐OH radical scavenging test; (C) ABTS radical scavenging test; (D) Clinical Radiotherapy and Space Radiation Therapeutics Through Reactive O_2_^−^ radical scavenging test. The graphs illustrate the concentration‐dependent scavenging effects of JAE and VC on DPPH, ‐OH, ABTS^+^, and O_2_^−^ radicals. Results are presented as mean ± SD (*n* = 6).

The radioprotective potential of botanical extracts is closely associated with their ability to mitigate radiation‐induced oxidative stress through free radical scavenging. In this study, we systematically evaluated the radical‐neutralizing capacity of JAE and found that it exhibited potent, concentration‐dependent antioxidant activity across multiple radical species (Figure 1). Specifically, JAE demonstrated notable efficacy in scavenging DPPH radicals, with a half maximal inhibitory concentration (IC_50_) of 1.38 ± 0.09 mg/mL, achieving a maximum scavenging rate of 93.96% ± 0.56% at 6.5 mg/mL (Figure 1A). The activity was even more pronounced against ABTS^+^ radicals, with an IC_50_ of 0.79 ± 0.041 mg/mL and a scavenging rate of 92.53% ± 0.12% at 1.5 mg/mL (Figure 1B). Remarkably, the strongest radical‐scavenging ability was observed against hydroxyl (‐OH) radicals, with an IC_50_ of only 0.6 ± 0.16 mg/mL (Figure 1C). In contrast, JAE exhibited relatively modest activity against superoxide anion (O_2_^−^) radicals, with an IC_50_ of 5 ± 1.3 mg/mL (Figure 1D). These findings highlight the radical‐selective antioxidant capacity of JAE. The extract demonstrated superior scavenging efficiencies against ABTS^+^ and ‐OH radicals compared to DPPH and O_2_^−^ radicals, reflecting a multi‐target, synergistic antioxidant mechanism that underpins its radioprotective effects.

### 3.2 Radioprotective Effects of JAE in X‐Ray Irradiated Mice

To further investigate the systemic radioprotective efficacy of JAE in vivo, a murine x‐ray irradiation model was established. During the 14‐day pre‐irradiation phase, all mice showed consistent weight gain. Following radiation exposure, a marked decline in body mass was observed across groups, with the model group experiencing a significantly greater rate of weight loss (1.30 g/day) compared to JAE‐treated mice (0.47 g/day). While the difference in post‐irradiation weight loss between the JAE and model groups did not reach statistical significance (*p* > 0.05), the attenuated decline in the JAE group nonetheless suggested a protective trend, indirectly supporting the potential radioprotective effects of JAE.

**FIGURE 2:**
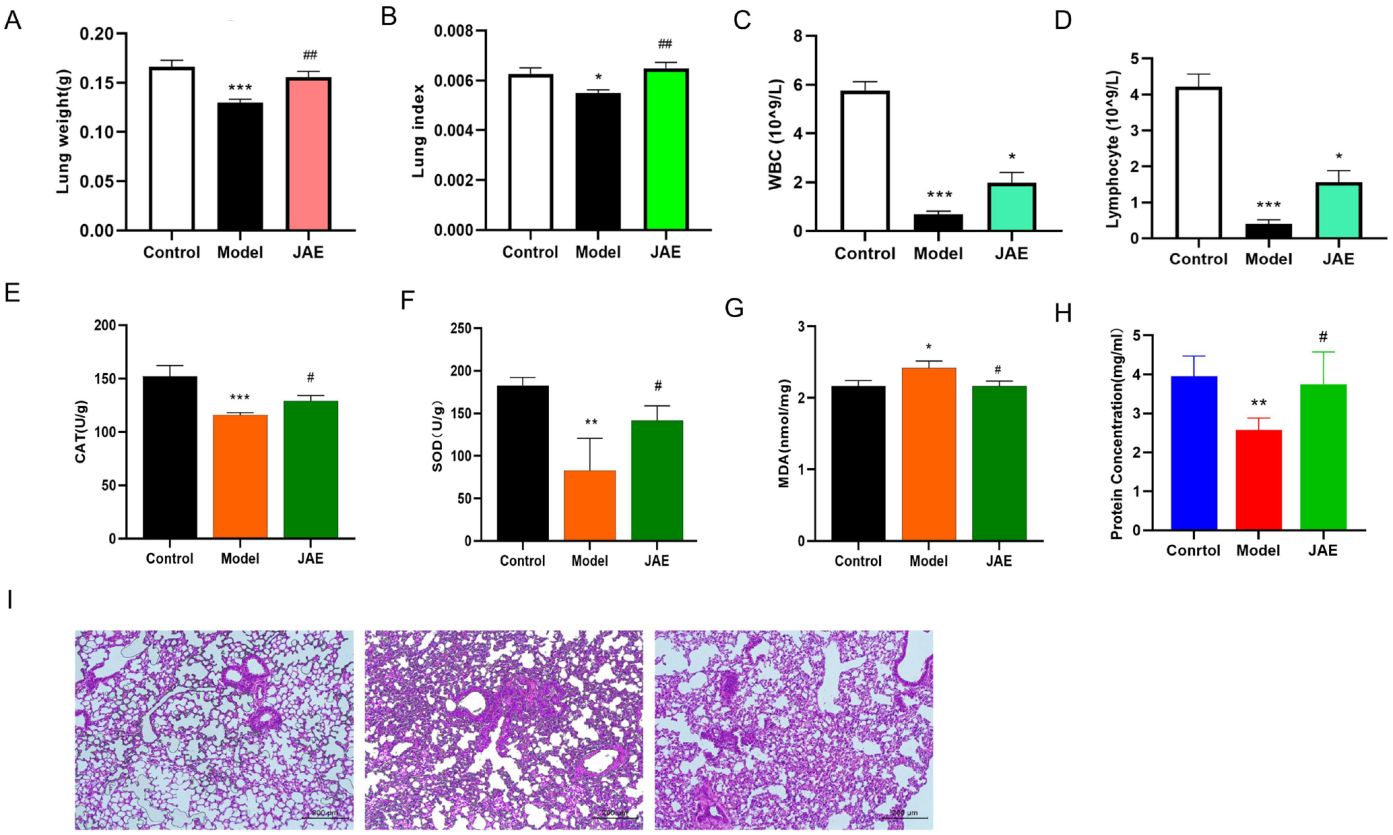
Effects of JAE on x‐ray mice. (A, B) The weight and index of lung in mice from control, model, and JAE groups. (C, D) Presents blood cell indices, including white blood cells, lymphocytes, in mice across groups. (E–G) Antioxidant‐related parameters: CAT activity (E), SOD activity (F), and MDA content (G) in lung tissue. (H) Displays protein content in lung tissue of control, model, and JAE groups. (I) Shows histopathological images of lung tissue via pathological staining of control, model, and JAE groups. Results are presented as mean ± SD (*n* = 6–8). *p < 0.05, **p < 0.01, ***p < 0.001 (vs. con group). #*p* < 0.05, ##*p* < 0.01, ###*p* < 0.001 (vs. UVC group).

Organ‐specific analysis revealed a selective protective effect of JAE on pulmonary tissues. No significant differences were noted in cardiac, hepatic, or splenic weights or organ indices (Figure S1A–C). However, JAE treatment markedly alleviated radiation‐induced lung damage. Compared to the irradiated model group, JAE‐treated mice increased by 16.51% in lung weight and a 15.16% improvement in lung index (organ‐to‐body weight ratio) (*p* < 0.05; Figure 2A,B), indicating preferential lung‐targeted efficacy. In addition to pulmonary protection, JAE demonstrated hematopoietic support by significantly reversing radiation‐induced leukopenia and lymphopenia. Peripheral leukocyte and lymphocyte counts increased 65.59% and 74.31%, respectively, in JAE‐treated mice versus the model group (*p* < 0.01; Figure 2C,D), highlighting its immuno‐rescue capabilities.

Biochemical analyses of lung tissue further substantiated JAE's antioxidative mechanisms. Treatment elevated CAT and SOD activities by 10.43% and 41.70%, respectively, while significantly reducing MDA levels by 13.59% compared to the model group (*p* < 0.05; Figure 2E–G). Histopathological evaluation further corroborated these findings, showing that JAE markedly alleviated radiation‐induced pulmonary congestion and interstitial edema while increasing total lung protein (*p* < 0.05; Figure 2H,I). Collectively, these results indicate that JAE confers organ‐selective radioprotection, particularly within the lung, offering targeted tissue resilience against radiation‐induced injury through enhancement of endogenous antioxidant defenses.

### 3.3 Systems Pharmacology Elucidation of JAE on the Proteome of Lung Tissue From Irradiated Mice

**FIGURE 3:**
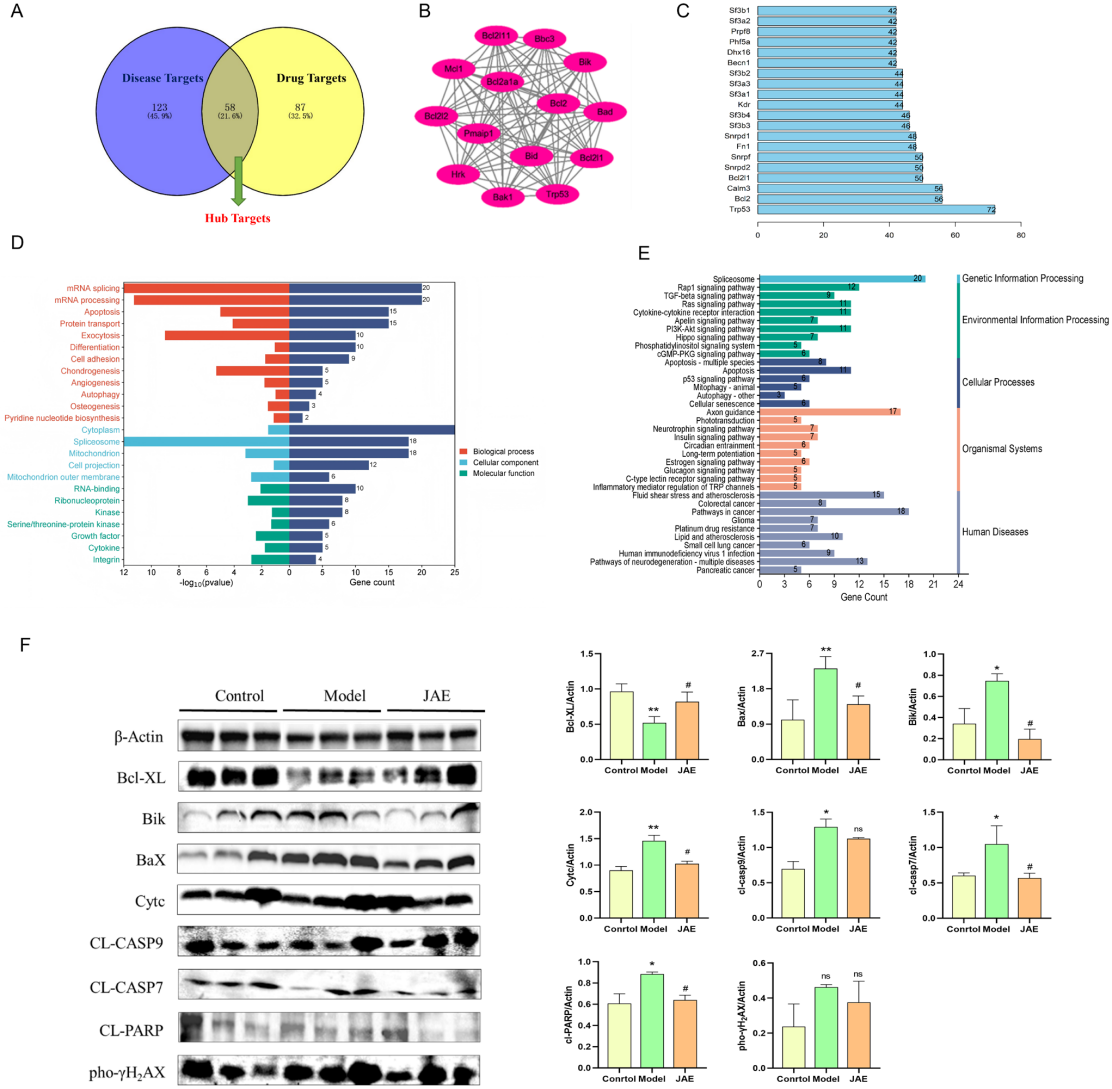
Network pharmacological analysis of JAE on the proteome of lung tissue from irradiated mice. (A) Venn diagram illustrating the overlap of DEPs among control, irradiated model, and JAE‐treated groups. (B) PPI network of 109 interacting proteins derived from STRING database analysis. (C) Topological screening prioritized 20 key targets based on degree centrality (> 15), predominantly enriched in cellular regulatory processes. (D, E) GO/KEGG co‐analysis showing significant enrichment of DEPs in cytoplasmic ribonucleoprotein complexes (*p* < 0.05) and apoptosis‐related pathways (*p* < 0.01). (F) Western blot analysis demonstrating JAE regulatory effects on Bcl‐family proteins and apoptosis/DNA damage‐related proteins. Results are presented as mean ± SD (*n* = 6–8). *p < 0.05, **p < 0.01, ***p < 0.001 (vs. con group). #*p* < 0.05, ##*p* < 0.01, ###*p* < 0.001 (vs. UVC group).

**FIGURE 4:**
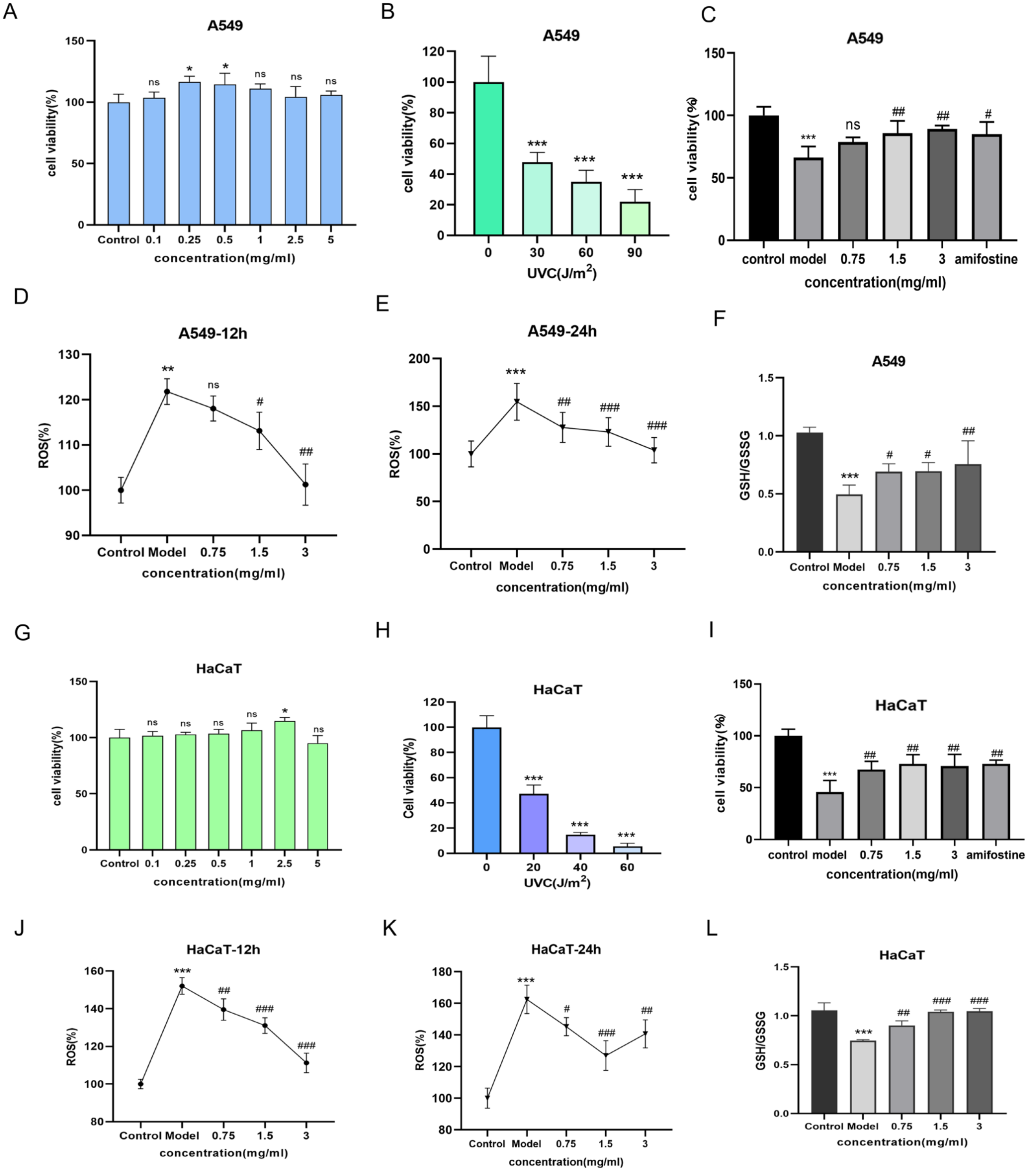
Effects of JAE on cell viability and UVC‐induced damage in A549 and HaCaT cells. (A) Cell viability of A549 cells treated with varying JAE concentrations to determine safe administration concentrations. (B) Cell viability of A549 cells exposed to different UVC irradiation energies. (C) Cell viability of A549 cells after UVC irradiation, following pre‐treatment with JAE at 0.75 mg/mL, 1.5 mg/mL, and 3 mg/mL. (D, E) ROS levels in A549 cells at 12 h and 24 h post‐UVC irradiation, detected via DCFH‐DA labeling after pre‐treatment with JAE (0.75 mg/mL, 1.5 mg/mL, 3 mg/mL) (F) GSH/GSSG levels in A549 cells post‐UVC irradiation, following pre‐treatment with gradient JAE concentrations. (G) Cell viability of HaCaT cells treated with varying JAE concentrations to determine safe administration concentrations. (H) Cell viability of HaCaT cells exposed to different UVC irradiation energies. (I) Cell viability of HaCaT and A549 cells after UVC irradiation, following pre‐treatment with JAE at 0.75 mg/mL, 1.5 mg/mL, and 3 mg/mL. (J, K) ROS levels in HaCaT cells at 12 h and 24 h post‐UVC irradiation, detected via DCFH‐DA labeling after pre‐treatment with JAE (0.75 mg/mL, 1.5 mg/mL, 3 mg/mL). (L) GSH/GSSG levels in HaCaT cells post‐UVC irradiation, following pre‐treatment with gradient JAE concentrations. Results are presented as mean ± SD (*n* = 3). *p < 0.05, **p < 0.01, ***p < 0.001 (vs. group). #*p* < 0.05, ##*p* < 0.01, ###*p* < 0.001 (vs. UVC group).

To systematically elucidate the molecular mechanisms underlying the radioprotective effects of JAE, we employed an integrative approach combining proteomic profiling with network pharmacology analysis (Figure 4). Using nano UHPLC–MS/MS, we identified differentially expressed proteins (DEPs) among the control, irradiated model, and JAE‐treated mouse lung tissues. A Venn diagram analysis revealed 58 core proteins associated with JAE‐mediated radioprotection (Figure 3A). PPI network analysis, constructed using the STRING database (Figure S2A), identified 109 interacting proteins, which were further analyzed using Cytoscape's MCODE plugin (parameters: Degree Cutoff = 2, Node Score Cutoff = 0.2, K‐Core = 2, Max. Depth = 100). This clustering identified critical functional modules and hub proteins (Figure 3B). Topological screening based on degree centrality (> 15) prioritized 20 key targets, which were predominantly enriched in cellular regulatory processes (Figure 3C). GO/KEGG co‐analysis demonstrated significant enrichment of in cytoplasmic ribonucleoprotein complexes (*p* < 0.05) and apoptosis‐related pathways (*p* < 0.01; Figure 3D,E). Consequently, differentially expressed genes (DEGs) were identified in the drug‐treated and model groups, based on the default threshold (| log2FC | > 2 and adjusted *P*‐value < 0.05) (Figure S3A,B).

Given JAE's demonstrated antioxidant properties and the established role of radiation‐induced oxidative stress in triggering DNA damage and apoptosis, we next explored whether JAE counteracts these effects by modulating mitochondrial apoptotic cascades. In irradiated mice, expression of the anti‐apoptotic protein Bcl‐XL was significantly downregulated, while pro‐apoptotic proteins Bax and Bik were upregulated (*p* < 0.05). Notably, JAE administration markedly reversed these effects, restoring Bcl‐XL expression and suppressing both Bax and Bik to near‐normal levels (*p* < 0.05). While radiation significantly elevated cytochrome c (Cty c) levels (*p* < 0.05), JAE did not significantly reduce this elevation (*p* > 0.05). Compared to the model group, JAE treatment did not exhibit any significant effect on cleaved caspase‐9 levels (*p* > 0.05), but resulted in a marked decrease in cleaved caspase‐7 expression. These findings suggest JAE selective inhibition of intrinsic apoptosis, suggesting its regulatory effects may primarily target caspase‐7 activation (Figure 3F). To assess DNA damage responses, we examined cleavage of poly (ADP‐ribose) polymerase (PARP) and phosphorylation of γ‐H2AX. Radiation significantly increased cleaved PARP levels (*p* < 0.05), which were effectively suppressed by JAE (*p* < 0.05). Although γ‐H2AX phosphorylation showed an increasing trend in the model group, it did not reach statistical significance (*p* > 0.05), and JAE treatment further reduced this phosphorylation without significant effect (*p* > 0.05). These findings suggest limited induction of DNA double‐strand breaks in this irradiation model and a partial protective effect of JAE on genomic integrity (Figure 3F).

Collectively, our proteomics evidence supports a synergistic radioprotective mechanism for JAE involving: (1) maintenance of transcriptional homeostasis, (2) inhibition of oxidative stress‐driven apoptosis, and (3) preservation of genomic integrity. These interconnected pathways likely contribute to JAE efficacy in mitigating radiation‐induced pulmonary damage and highlight its potential as a systemic radioprotective agent with tissue‐specific activity.

### 3.4 Radioprotective Effects of JAE in UVC‐Induced Cellular Damage

Following the confirmation of JAE's protective effects against pulmonary damage in vivo, we established in vitro injury models to elucidate its cellular mechanisms. For lung‐specific evaluation, A549 human pulmonary adenocarcinoma cells were utilized. Cytotoxicity screening demonstrated JAE biocompatibility (0.1–5 mg/mL), with optimal A549 proliferation observed at lower concentrations (0.25–0.5 mg/mL; Figure 4A). Three concentrations (0.75, 1.5, and 3 mg/mL) were selected for radioprotection assays. UVC dose–response analyses identified 30 J/m^2^ as the threshold dose for A549 cells, reducing viability by 50% (Figure 4B). Pretreatment with 3 mg/mL JAE significantly restored viability, underscoring its dual role in enhancing proliferation and counteracting UVC cytotoxicity. 3 mg/mL JAE demonstrated superior radioprotective efficacy against UVC radiation compared to amifostine (Figure 4C). JAE mitigated UVC‐induced oxidative stress in A549 cells by suppressing ROS accumulation and restoring glutathione homeostasis. At 12 h post‐irradiation, ROS levels increased by 21.80% (*p* < 0.05), with mid‐ and high‐dose JAE (1.5 and 3 mg/mL) significantly reducing ROS (*p* < 0.05). By 24 h, all JAE concentrations markedly lowered ROS levels (low‐, mid‐, and high‐dose JAE: 17.40%, 20.47%, and 32.88%, respectively; *p* < 0.05) (Figure 4D,E). UVC irradiation also disrupted glutathione balance, significantly decreasing the GSH/GSSG ratio (*p* < 0.05). JAE pretreatment reversed this effect, restoring redox equilibrium in a dose‐dependent manner (Figure 4F).

Cutaneous damage remains a major concern due to the skin's pivotal role as the first line of defense against environmental radiation, particularly UV exposure. To evaluate the radioprotective efficacy of JAE in epidermal cells, a parallel set of experiments was conducted in HaCaT keratinocytes using an optimized UVC‐induced injury model. Cytotoxicity profiling revealed maximal HaCaT viability at 2.5 mg/mL JAE (Figure 4G), prompting the use of 0.75–3 mg/mL for subsequent assays. UVC exposure at 20 J/m^2^ reduced HaCaT viability by 50% (Figure 4H), with optimal protection observed at 1.5 mg/mL JAE. Their protective effects were comparable to those of amifostine (Figure 4I). UVC‐induced oxidative stress elevated ROS levels to 52.08% (12 h) and 62.47% (24 h; *p* < 0.05). JAE pretreatment reduced ROS by 35.52% (1.5 mg/mL, 12 h) and 21.79% (3 mg/mL, 24 h; *p* < 0.05; Figure 4J,K). Additionally, JAE restored the GSH/GSSG ratio dose‐dependently (0.75–3 mg/mL; *p* < 0.05), reestablishing redox homeostasis in keratinocytes (Figure 4L). These results underscore the ability of JAE to suppress ROS accumulation and restore intracellular redox balance, highlighting its potential as a cell type‐ and time‐dependent antioxidant agent against UVC‐induced oxidative stress.

### 3.5 Arbutin Confers Radioprotection in HaCaT Cells via Antioxidant Pathways

**FIGURE 5:**
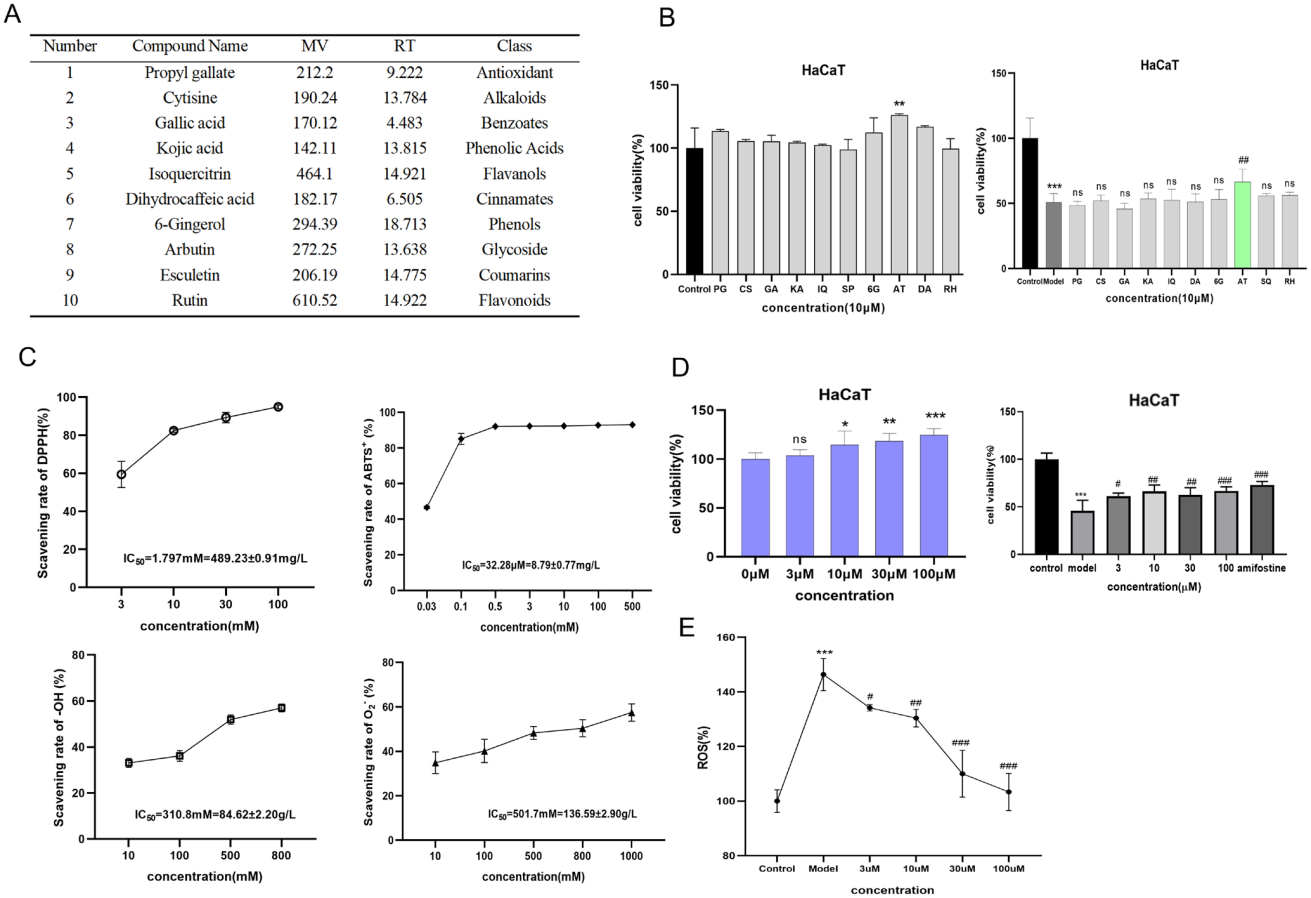
Antioxidant activity of arbutin on UVC‐damaged cells. (A) Identification of 10 compounds from JAE via LC–MS screening. (B) Cell viability analysis of HaCaT cells treated with 10 compounds, evaluating radioprotective effects. (C) Antioxidant activity assays of Arbutin, showing DPPH, O_2_^−^, OH, and ABTS radical scavenging rates across different concentrations. (D) Cell viability of HaCaT cells under various Arbutin concentrations, including normal and UVC‐irradiated groups. (E) ROS levels in HaCaT cells pre‐treated with different Arbutin concentrations post‐UVC irradiation. Results are presented as mean ± SD (*n* = 3). *p < 0.05, **p < 0.01, ***p < 0.001 (vs. con group). #*p* < 0.05, ##*p* < 0.01, ###*p* < 0.001 (vs. UVC group).

LC–MS‐based metabolomic analysis of JAE identified 10 natural compounds with anti‐inflammatory, antioxidant, and photoprotective properties, which were subsequently subjected to preliminary in vitro screening (Figure 5A). Among these, arbutin emerged as the most promising candidate, demonstrating robust and reproducible anti‐radiation efficacy in HaCaT cell models.

In initial cytotoxicity screening of 10 compounds, arbutin demonstrated dose‐dependent cytoproliferative effects on HaCaT cells. Under UVC‐induced radiation stress (20 J/m^2^ for HaCaT), arbutin revealed exceptional radioprotective efficacy at 10 μM, significantly enhancing HaCaT viability by 31.55% (*p* < 0.05) (Figure 5B). Notably, arbutin's antioxidant capacity was concentration‐dependent, achieving > 90% ABTS^+^ radical scavenging across a broad range (0.5–500 μM), far surpassing its modest DPPH radical neutralization (46.61% at 30 μM) (Figure 5C). This selectivity, likely attributed to its phenolic glycoside structure, underscores its mechanism‐specific radical scavenging behavior. Compared with the untreated control (0 μM), arbutin treatment at 10, 30, and 100 μM significantly enhanced cell viability (*p* < 0.05), while 3 μM showed no statistical significance (*p* > 0.05). Under UVC‐induced radiation stress (20 J/m^2^), arbutin administration at 3, 10, 30, and 100 μM achieved statistically significant improvements in cellular viability relative to irradiated controls, with respective increases of 26.61%, 33.34%, 42.08%, and 68.02%. The effects of 100 μM arbutin were comparable to that of amifostine (*p* < 0.05, Figure 5D). Pretreatment with 30 μM and 100 μM arbutin reduced UVC‐induced ROS levels in HaCaT cells by 42.3% and 58.7% (Figure 5E). The present study reveals the molecular mechanism of arbutin through multi‐target synergistic protection against UVC radiation damage by combining LC–MS and experimental validation, which provides a theoretical basis for its application in the radiation protection field.

### 3.6 Multi‐Dimensional Protection of Arbutin Through Cell Cycle Regulation and Mitochondrial Preservation

**FIGURE 6:**
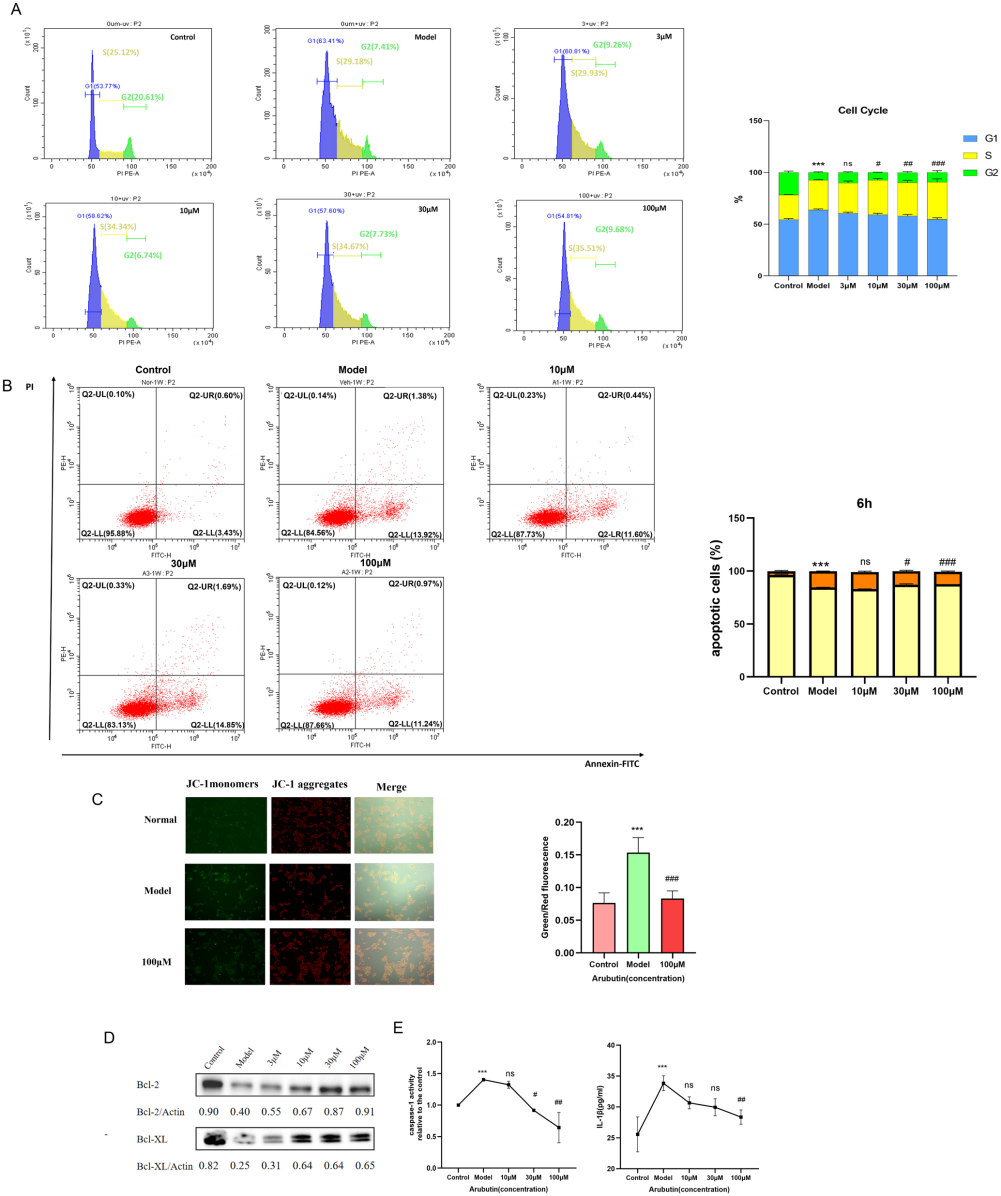
Arbutin alleviates UVC‐induced radiative damage via cell cycle regulation, apoptosis inhibition, and mitochondrial protection in HaCaT cells. (A) Flow cytometry analysis of cell cycle phases (G1, S, G2) in HaCaT cells under control, UVC‐irradiated model, and arbutin (3 μM, 10 μM, 30 μM, 100 μM) combined with UVC treatments. (B) Cell apoptosis analysis of HaCaT cells pre‐treated with arbutin (10 μM, 30 μM, 100 μM) detected by Annexin V/PI double staining at 6 h post‐UVC irradiation. (C) Mitochondrial membrane potential evaluation using JC‐1 probe in HaCaT cells across different treatment groups. (D) Western blot analysis of Bcl‐2 and Bcl‐XL protein expression in HaCaT cells with various treatments. (E) Detection of Caspase 1 activity and IL‐1β content in HaCaT cells after control, model, arbutin (10 μM, 30 μM, 100 μM), or positive control treatments. Results are presented as mean ± SD (*n* = 3). *p < 0.05, **p < 0.01, ***p < 0.001 (vs. con group). #*p* < 0.05, ##*p* < 0.01, ###*p* < 0.001 (vs. CONUVC group).

We systematically investigated the protective effects of arbutin on UVC‐induced DNA damage and apoptosis in HaCaT cells and its mechanism. Firstly, cell cycle analysis revealed that UVC irradiation led to a significant increase (*p* < 0.05) in the proportion of cells in the G1 phase and S phase of HaCaT cells (Figure 6A), and a decrease in the proportion of cells in the G2 phase, suggesting that UVC induced a cell cycle block in the G1/S phase; the medium‐high concentration of arbutin (30–100 μM) could dose‐dependently alleviate this blocking effect, suggesting that it promotes DNA damage repair by maintaining normal cell cycle progression (Figure 6).

Apoptosis was further detected by Annexin V/PI double staining combined with flow cytometry, and the results showed that the apoptosis rate in the model group was significantly increased after 6 h of UVC irradiation, and both 30 μM and 100 μM arbutin showed significant inhibitory effects (2.63% and 3.86% reduction, respectively). By 18 h, apoptosis rose to 29.84% ± 1.66%, where arbutin (10–100 μM) exhibited graded inhibition (3.41%–16.31%, *p <* 0.05). At 24 h peak apoptosis (48.16% ± 1.15%), 100 μM arbutin (12.53%, *p <* 0.05). 10 μM arbutin remained ineffective throughout (*p >* 0.05), suggesting that its anti‐apoptotic effects were time‐ and dose‐dependent (Figure 6B and Figure S4A). Mechanistic studies showed that 30 μM arbutin significantly improved the mitochondrial membrane potential of UVC‐injured cells (Figure 6C) and inhibited the mitochondria‐dependent apoptotic pathway by up‐regulating the expression of anti‐apoptotic proteins Bcl‐2 and Bcl‐XL (*p* < 0.01; Figure 6D). Arbutin demonstrated t inhibition of UVC‐induced Caspase‐1 activity significant at 30 μM (*p* < 0.05) and IL‐1β production reduced by 100 μΜ (*p* < 0.05; Figure 6E). Arbutin suppresses UVC‐triggered pyroptosis and exhibits anti‐inflammatory activity by targeting Caspase‐1/IL‐1β signaling. These results suggest that arbutin exerts its protective effect against UVC injury through multi‐targeted actions—including maintenance of cell cycle homeostasis, protection of mitochondrial function, and modulation of apoptosis‐related protein expression‐collectively.

## 4 Discussion

Radiation exposure poses a critical threat to human health, causing cellular and tissue injury through mechanisms including oxidative stress, DNA damage, and mitochondrial dysfunction. These pathological effects can ultimately contribute to fibrosis, carcinogenesis, and multi‐organ failure (Geisler et al. 2021; Niu et al. 2023). Currently available radioprotective agents, such as amifostine and various synthetic antioxidants, are limited by systemic toxicity and a narrow mechanistic focus, typically targeting only a single pathway, such as ROS scavenging or DNA repair (Ji et al. 2023; Shimura 2023). In contrast, the present study demonstrates that JAE exhibits significant radioprotective properties through multifaceted mechanisms. Proteomic analysis revealed that JAE mitigates x‐ray‐induced lung injury in mice by modulating apoptosis and oxidative stress pathways. In vitro experiments confirmed its efficacy in protecting A549 lung cells and HaCaT keratinocytes against UVC radiation, underscoring its broad‐spectrum cellular protection. LC–MS identified arbutin as a major bioactive constituent of JAE, which was shown to counteract UVC‐induced damage in HaCaT cells. Mechanistically, arbutin protects against UVC‐induced cellular damage primarily by suppressing apoptotic signaling and enhancing antioxidant defenses, thereby offering dual therapeutic targeting of radiation‐induced oxidative stress and cell death.

Radiation, whether ionizing (x/*γ*‐rays) or non‐ionizing (UVC), disrupts redox homeostasis through both direct and indirect mechanisms. Direct ionization of cellular water molecules leads to the generation of ROS, including ‐OH and O_2_^−^, while indirect effects impair antioxidant enzyme activity (Ping et al. 2020; Wei, Wang, Wang, Meng, et al. 2019). These ROS saturate endogenous defenses, including superoxide dismutase (SOD), catalase (CAT), glutathione peroxidase (GPx), and the GSH/GSSG redox buffer. The excessive ROS production irreversibly inactivates SOD (which converts O_2_^−^ to H_2_O_2_) and CAT/GPx (which detoxify H_2_O_2_), creating a self‐amplifying cycle of oxidative stress. This cascade depletes reduced glutathione (GSH), elevates oxidized glutathione (GSSG), and disrupts redox homeostasis, ultimately triggering lipid peroxidation, DNA damage, and mitochondrial apoptosis (Köroğlu et al. 2024; Özturk et al. 2024). Tissues with high oxygen tension and proliferative activity, such as the lungs and skin, are particularly susceptible to radiation‐induced oxidative injury. In the lung, the extensive alveolar surface area facilitates oxygen diffusion but simultaneously magnifies oxidative stress cascades, thereby accelerating inflammation, fibrosis, and apoptosis (Guo et al. 2023; Zhu et al. 2021). JAE emerges as a potent radioprotective agent by directly scavenging ROS and restoring redox balance. In vitro studies demonstrated JAE's superior ABTS^+^ radical scavenging capacity. In UVC‐irradiated HaCaT keratinocytes, JAE significantly restored the GSH/GSSG ratio. Unlike agents targeting a single oxidative pathway, JAE exerts a multi‐faceted protective effect by combining ROS scavenging, GSH regeneration, and mitochondrial stabilization. This combinatorial mechanism positions JAE as a holistic and mechanistically versatile countermeasure against radiation‐induced cellular injury.

JAE demonstrates significant therapeutic potential in alleviating radiation‐induced lung injury by targeting mitochondrial dysfunction and apoptosis. Radiation‐induced mitochondrial dysfunction triggers apoptosis via Bcl‐2/Bax imbalance and caspase‐3 activation (Cui et al. 2023; Ogura et al. 2009). JAE restores the Bcl‐2/Bax ratio toward anti‐apoptotic dominance, suppressing caspase‐3 cleavage, and stabilizing mitochondrial membranes, thereby blocking cytochrome c efflux and halting the apoptotic cascade. Recent evidence highlights bidirectional crosstalk between the Nrf2‐mediated antioxidant pathway and the Bcl‐2 anti‐apoptotic network; for instance, Nrf2 can transactivate BCL2 expression through antioxidant response elements (AREs), while Bcl‐2 in turn stabilizes Nrf2 by attenuating mitochondrial ROS (Niture and Jaiswal 2013). Natural products such as *Inula britannica* polyphenols and curcumin have been shown to exploit this synergy, simultaneously activating Nrf2 and elevating Bcl‐2 to achieve significantly greater cytoprotection than targeting either pathway alone (Shahcheraghi et al. 2021; Wu et al. 2023). Although our proteomic and network analyses suggest that JAE modulates antioxidant and anti‐apoptotic pathways, the synergistic or additive effects between these pathways were not quantitatively evaluated in the current study. Future studies employing combinatorial pathway inhibition will be essential to quantitatively dissect the cooperative effects underlying JAE's multi‐target radioprotection. In murine models, JAE reduced radiation‐induced lung weight loss and improved the lung index. This lung‐specific protection underscores the heightened oxidative microenvironment of pulmonary tissue post‐irradiation, which appears to potentiate JAE's antioxidant and anti‐apoptotic activities. By stabilizing mitochondrial integrity, JAE prevents energy collapse and preserves cellular viability, offering a critical advantage over agents lacking mitochondrial targeting.

Radiation significantly impairs hematopoietic function by inducing DNA strand breaks, oxidative stress, and apoptotic cascades, with lymphocytes exhibiting heightened vulnerability to these cytotoxic insults (Byun et al. 2021; Venkatesulu et al. 2022). Clinically, this manifests as acute lymphopenia and systemic immunosuppression, characterized by the depletion of memory B‐cell reservoirs, dysregulation of T‐cell homeostasis, and suppression of pro‐inflammatory cytokine production. These effects are further exacerbated when lymphoid‐rich tissues or circulating immune cells are directly irradiated, contributing to adverse clinical outcomes and prolonged immunosuppressive states (Ghajar‐Rahimi et al. 2025; Hart and Norval 2021). Beyond its established antioxidant and anti‐apoptotic properties, JAE exhibits potent immunomodulatory effects critical for comprehensive radioprotection (Feng et al. 2025). Experimental data demonstrate that JAE facilitates lymphocyte recovery in irradiated models through coordinated mechanisms: restoration of hematopoietic integrity is paralleled by the re‐establishment of redox balance, thereby attenuating acute oxidative injury and mitigating long‐term immune dysfunction. This multifaceted pharmacological profile, combining immunostimulatory, cytoprotective, and antioxidant pathways, positions JAE as a promising therapeutic candidate for total‐body irradiation scenarios where concurrent protection against immediate cellular damage and long‐term immune compromise is clinically imperative.

Arbutin, identified as a pivotal constituent of JAE through comprehensive metabolomic profiling of jujube, was selected from a pool of 10 candidate bioactive compounds (including flavonoids, phenolic acids, and triterpenoids) based on its anti‐UVC radiation protective activity (Chen et al. 2021). Like ginsenosides, EGCG, and curcumin, it exerts radioprotective effects predominantly through this antioxidant pathway. However, their limited influence on inflammation‐associated signaling, particularly inflammasome activation, highlights a mechanistic shortcoming (Sumiyoshi and Kimura 2009; Tang et al. 2018; Xie et al. 2020). Notably, radiation exposure not only disrupts redox homeostasis but also activates the NLRP3 inflammasome, driving caspase‐1‐dependent IL‐1β maturation and pyroptosis (Rao et al. 2023). While EGCG and similar agents enhance Nrf2‐driven antioxidant capacity, their minimal suppression of inflammasome‐associated markers such as IL‐1β stands in stark contrast to the dual‐action profile of arbutin (Xie et al. 2020). Arbutin not only restores GSH expression but also significantly downregulates caspase‐1 activation and IL‐1β secretion. This dual‐targeting mechanism, absent in single‐pathway agents, may offer superior protection in radiation exposure contexts where radiation triggers parallel ROS and inflammasome activation, a phenomenon increasingly reported in high‐dose or chronic exposure models (Wei, Wang, Wang, Wang, et al. 2019). These findings underscore the need to reevaluate conventional radioprotection strategies, advocating for integrated targeting of antioxidant and anti‐inflammatory pathways.

We fully recognize that this study is subject to several limitations. Firstly, while the present study demonstrates the strong radioprotective efficacy of JAE, its potential application necessitates a thorough evaluation of long‐term safety and pharmacokinetic properties, which remains a limitation of our current work. It is important to note that extracts from *Ziziphus jujuba* Mill. have a well‐documented history of use in traditional medicine and are generally recognized for their safety profile (Rodríguez Villanueva and Rodríguez Villanueva 2017). Several studies have reported on the low toxicity and favorable safety of jujube extracts in rodent models (Alsayari and Wahab 2021). However, comprehensive long‐term toxicological data, detailed absorption, distribution, metabolism, and excretion (ADME) profiles, and potential off‐target effects specific to the context of radioprotection are still largely unexplored. Therefore, future studies will be essential to delineate the chronic toxicity, organ‐specific clearance, and optimal dosing regimens of JAE to fully assess its viability for human use as a radioprotective agent.

Secondly, the UV‐C and X‐ray irradiation models applied here, while well‐established for simulating certain types of Earth‐based radiation exposure (e.g., medical or environmental) (Graupner et al. 2017), do not fully represent the complex mixed‐radiation spectrum characteristic of the aerospace environment (Cucinotta and Durante 2006). Nevertheless, findings derived from the model provide valuable mechanistic insights and a foundational assessment of JAE's radioprotective potential, which can inform future research aimed at developing countermeasures against space radiation. Subsequent studies will incorporate more physiologically relevant radiation types, such as heavy ions, to better simulate cosmic radiation effects. Furthermore, we recognize the potential differences in metabolism and bioavailability between murine models and humans (Martignoni et al. 2006). To better predict human responses, future work will evaluate the pharmacokinetics and efficacy of JAE in large animal models phylogenetically closer to humans, such as beagles or cynomolgus monkeys.

## 5 Conclusion and Outlook

This study demonstrates that the radioprotective effects of JAE against radiation‐induced lung and cellular damage involve arbutin‐mediated dual suppression of cell death via oxidative stress pathway modulation. As a novel food‐derived radioprotectant, JAE offers a promising therapeutic avenue; however, several challenges remain. The possibility of synergistic effects of multiple compounds in JAE beyond arbutin should be noted. It should be noted that although arbutin was identified as the core bioactive compound mediating JAE's in vitro effects, its in vivo efficacy and pharmacokinetic profile (e.g., bioavailability and tissue distribution) remain unvalidated. Efficacy varies across radiation types (UVC vs. x/*γ*‐rays) due to differing ROS generation mechanisms, necessitating type‐specific formulations. Future studies should prioritize in vivo validation of arbutin and detailed pharmacokinetic analysis, alongside AI‐driven bioinformatics and multi‐omics approaches to optimize processing and dose–response relationships (Tong et al. 2024). Clinical validation is essential to translate these findings into therapies. Addressing these gaps could enable JAE‐based interventions to redefine radioprotection strategies, integrating traditional medicine with modern pharmacology.

## Author Contributions

**Liping Wang:** data curation (equal), formal analysis (equal), investigation (equal), writing – original draft (lead), writing – review and editing (equal). **Hui Yang:** data curation (equal), investigation (equal), writing – original draft (equal). **Xuchen Zhou:** investigation (equal), methodology (equal). **Long Li:** methodology (equal), writing – review and editing (equal).

## Conflicts of Interest

The authors declare no conflicts of interest.

## Supporting information


**Figure S1:** Physiological alterations in mice pre‐ and post‐X‐ray irradiation: (A) Body weight dynamics; (B) Absolute organ mass (spleen, thymus, liver, kidneys); (C) Organ index. Results are presented as mean ± SD (*n* = 6–8). *p < 0.05, **p < 0.01, ***p < 0.001 (vs. con group). #*p* < 0.05, ##*p* < 0.01, ###*p* < 0.001 (vs. UVC group).
**Figure S2:** Protein–protein interaction (PPI) network analysis.
**Figure S3:** Differential gene expression analysis in the model versus drug‐treated groups. (A) Volcano plot illustrating significantly dysregulated genes. (B) Heatmap displaying clustered expression patterns of differentially expressed genes (DEGs). DEGs were identified using thresholds of |log2FC| > 2 and adjusted *p*‐value < 0.05.
**Figure S4:** Cell apoptosis analysis of HaCaT cells pre‐treated with arbutin (10 μM, 30 μM, 100 μM) detected by Annexin V/PI double staining at 18 h and 24 h post‐UVC irradiation. Results are presented as mean ± SD (*n* = 3). *p < 0.05, **p < 0.01, ***p < 0.001 (vs. con group). #*p* < 0.05, ##*p* < 0.01, ###*p* < 0.001 (vs. UVC group).

## Data Availability

The data that support the findings of this study are available from the corresponding author upon reasonable request.
