# Supplementary material for: Radioprotective Efficacy of Jujube Aqueous Extract and Arbutin: Synergistic Antioxidant and Anti‐Apoptotic Mechanisms in Radiation Injury
Source: Food Sci Nutr. 2025 Oct 23;13(10):e71073. doi: 10.1002/fsn3.71073 (PMC12547836; doi:10.1002/fsn3.71073)
Supplement: Supplementary file 1 — Figure S1: Physiological alterations in mice pre‐ and post‐X‐ray irradiation: (A) Body weight dynamics; (B) Absolute organ mass (spleen, thymus, liver, kidneys); (C) Organ index. Results are presented as mean ± SD (n = 6–8). *p < 0.05, **p < 0.01, ***p < 0.001 (vs. con group). #p < 0.05, ##p < 0.01, ###p < 0.001 (vs. UVC group). Figure S2: Protein–protein interaction (PPI) network analysis. Figure S3: Differential gene expression analysis in the model versus drug‐treated groups. (A) Volcano plot illustrating significantly dysregulated genes. (B) Heatmap displaying clustered expression patterns of differentially expressed genes (DEGs). DEGs were identified using thresholds of |log2FC| > 2 and adjusted p‐value < 0.05. Figure S4: Cell apoptosis analysis of HaCaT cells pre‐treated with arbutin (10 μM, 30 μM, 100 μM) detected by Annexin V/PI double staining at 18 h and 24 h post‐UVC irradiation. Results are presented as mean ± SD (n = 3). *p < 0.05, **p < 0.01, ***p < 0.001 (vs. con group). #p < 0.05, ##p < 0.01, ###p < 0.001 (vs. UVC group). [file FSN3-13-e71073-s001.docx]

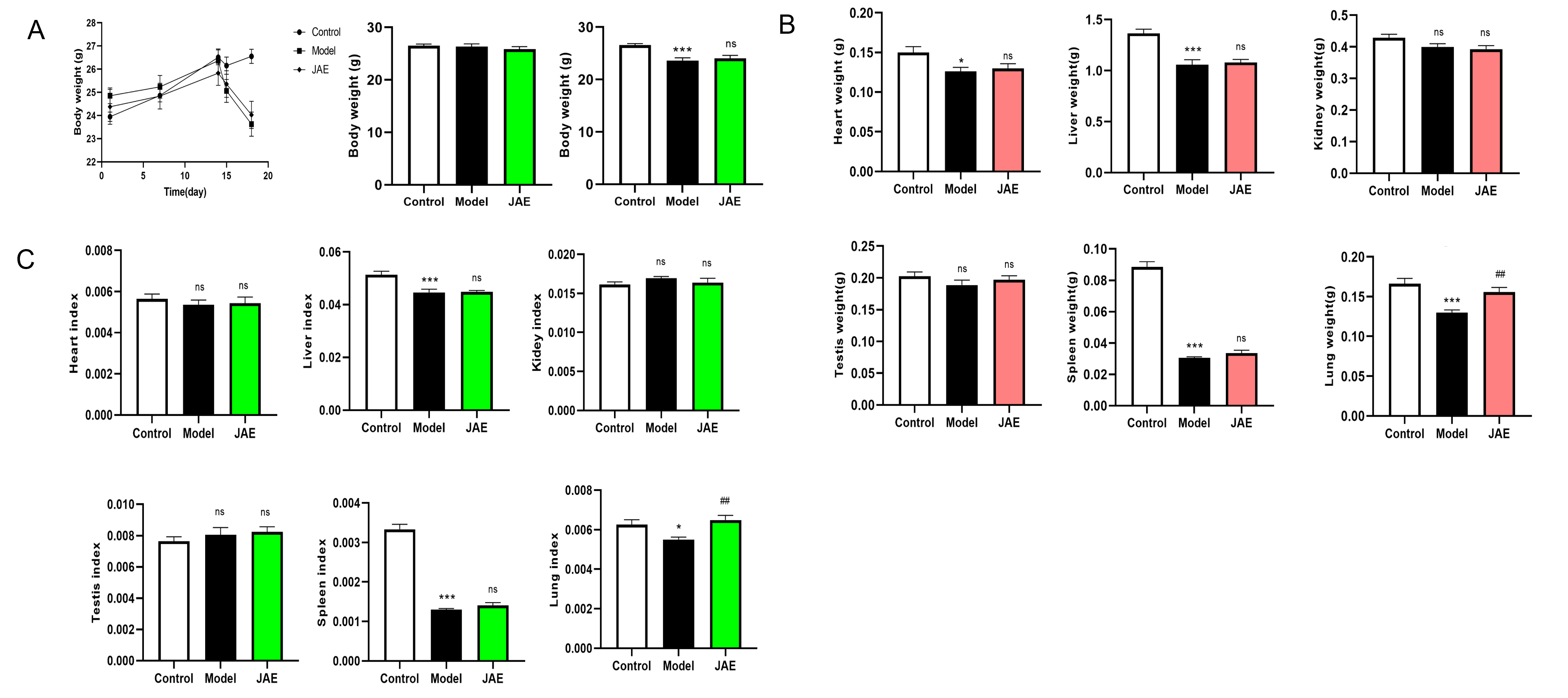


**Figure S1** Physiological alterations in mice pre - and post-X-ray irradiation: (A) Body weight dynamics; (B) Absolute organ mass (spleen, thymus, liver, kidneys); (C) Organ index.Results are presented as mean ± SD (n = 6-8). **P* < 0.05, ***P* < 0.01, ****P* < 0.001 (vs. con group). #*P* < 0.05, ##*P* < 0.01, ###*P* < 0.001 (vs. UVC group).


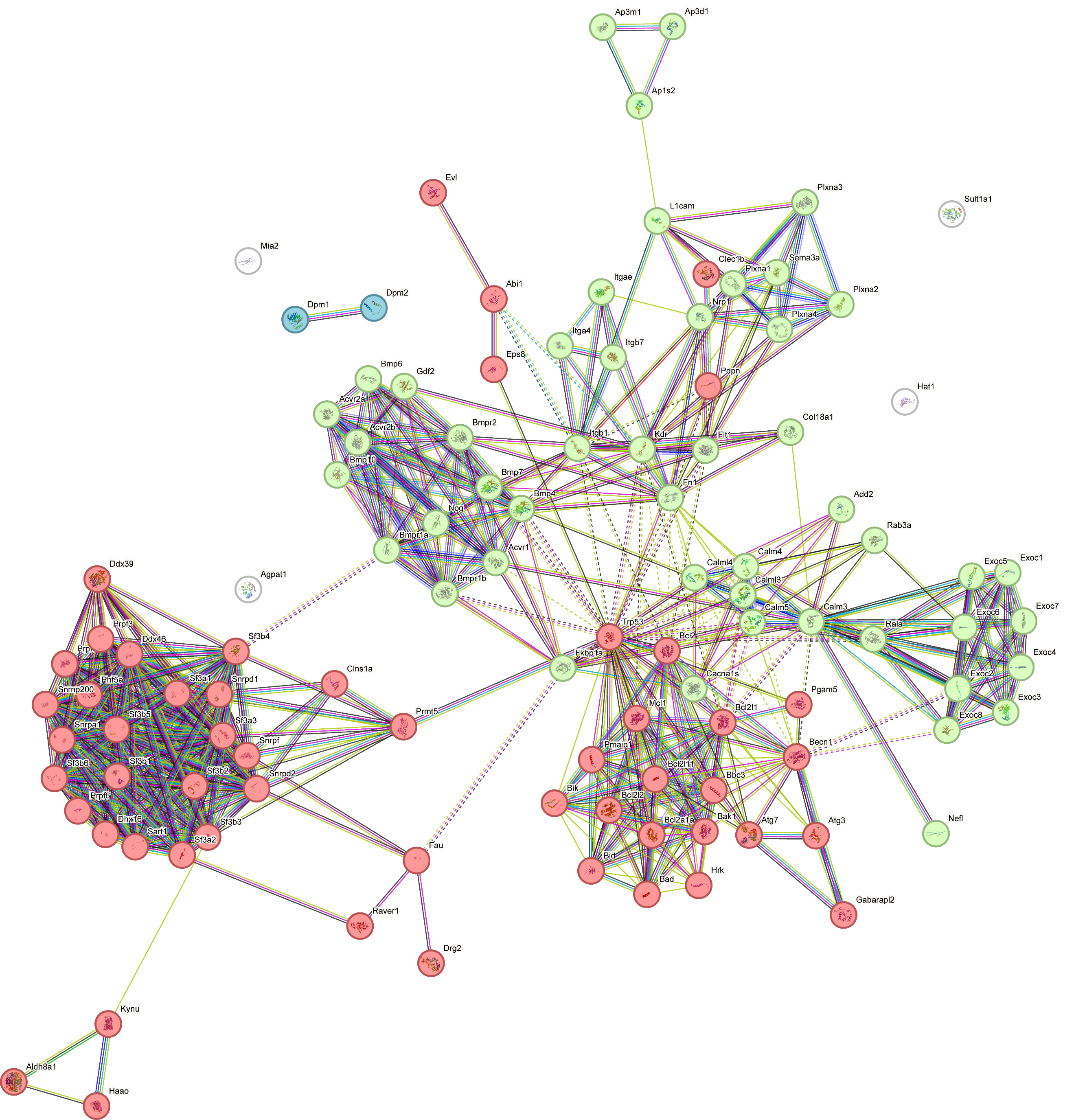


**Figure S2** Protein-protein interaction (PPI) network analysis.

**
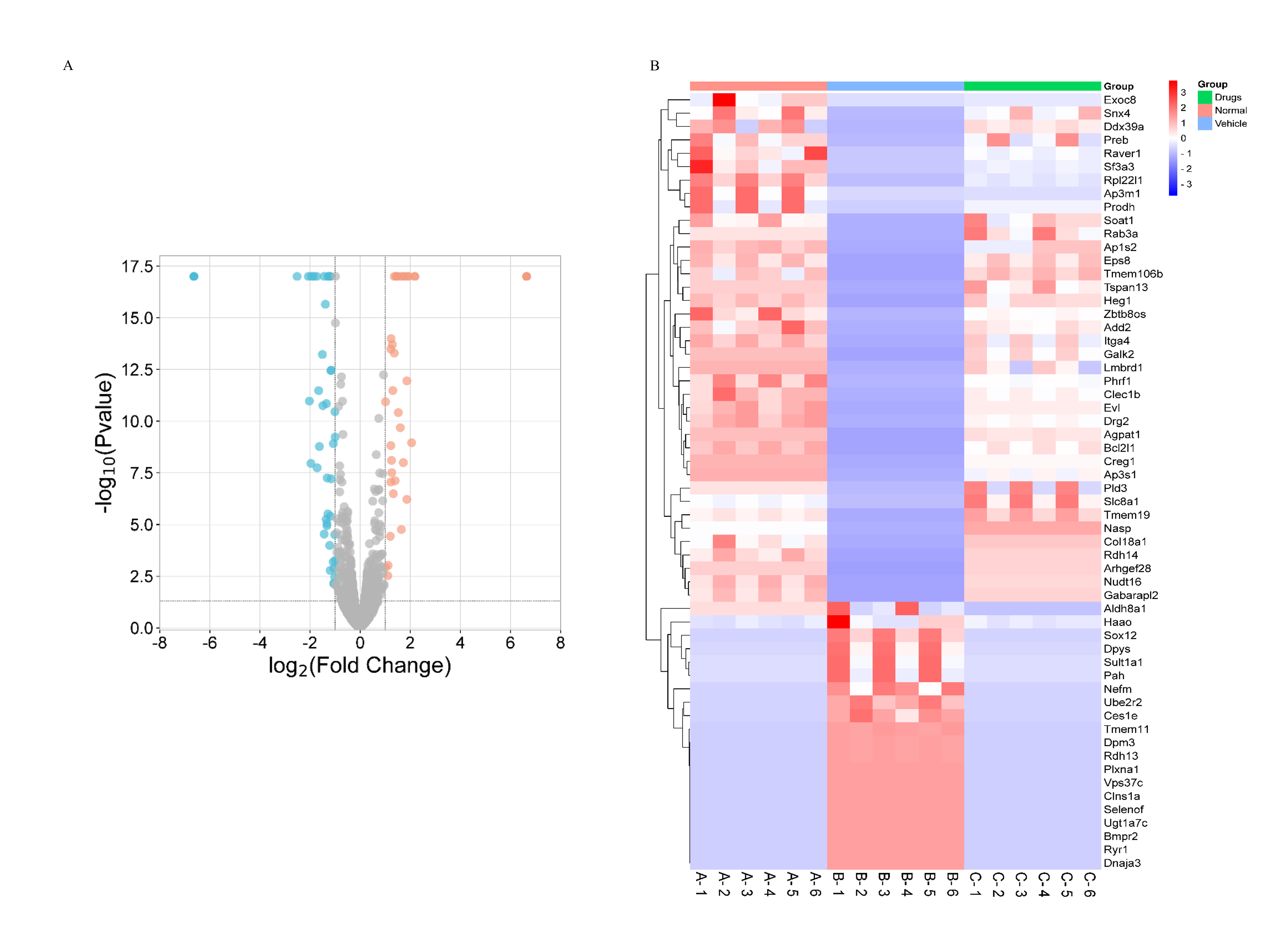
**

Figure S3. Differential gene expression analysis in the model versus drug-treated groups. (A) Volcano plot illustrating significantly dysregulated genes. (B) Heatmap displaying clustered expression patterns of differentially expressed genes (DEGs). DEGs were identified using thresholds of |log2FC| > 2 and adjusted *P*-value < 0.05.

**
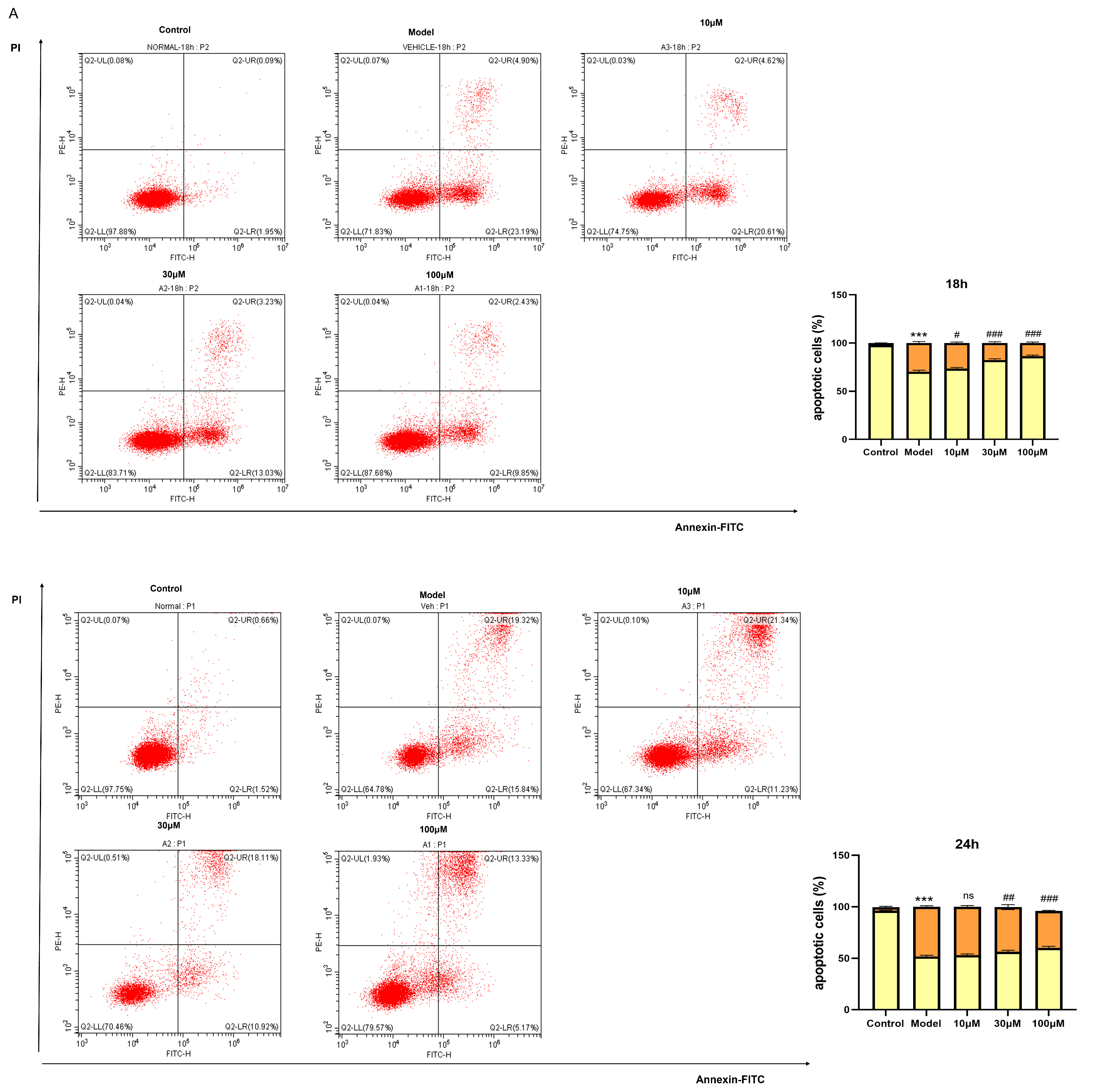
**

**Figure S4** Cell apoptosis analysis of HaCaT cells pre-treated with arbutin (10μM, 30μM, 100μM) detected by Annexin V/PI double staining at 18h and 24h post-UVC irradiation. Results are presented as mean ± SD (n = 3). **P* < 0.05, ***P* < 0.01, ****P* < 0.001 (vs. con group). #*P* < 0.05, ##*P* < 0.01, ###*P* < 0.001 (vs. UVC group).
